# T1 rhizotomy for post-stroke hand flexion spasticity: a preliminary report of 15 cases

**DOI:** 10.3389/fsurg.2026.1860760

**Published:** 2026-06-17

**Authors:** Guang-Hui Gao, Wen-De Wang, Zuo-Bin Hao, Xing-Jie Gao, Yun-Li Zhou, Ye-Ben Wang, Sheng-Jun Duan

**Affiliations:** Jinan Third People's Hospital, Jinan, China

**Keywords:** central limb spasticity, hand flexion spasticity, muscle tone reduction, stroke, T1 rhizotomy

## Abstract

**Background and objective:**

Stroke is the leading cause of central upper limb spastic paralysis, with hand flexion spasticity being the most common and functionally debilitating manifestation. Various surgical approaches have been explored, yet optimal management remains challenging. This study proposes T1 rhizotomy as a novel surgical intervention for post-stroke hand flexion spasticity.

**Methods:**

Fifteen consecutive patients with unilateral post-stroke hand flexion spasticity underwent T1 rhizotomy between November 2023 and December 2024. Under general anesthesia, the T1 nerve root was exposed via a supraclavicular approach, transected distally, and a 1 cm proximal segment was resected. Safety and complications were recorded. Patients were followed for at least one year. Spasticity was assessed using the Modified Ashworth Scale (MAS) and hand function using the modified House Functional Classification (HFC). Functional and ADL outcomes were assessed using the QuickDASH questionnaire and the Barthel Index.

**Results:**

All procedures were completed successfully without intraoperative complications or major adverse events. Transient postoperative numbness or mild forearm pain occurred in some patients and resolved within three months. At final follow-up, mean MAS score decreased from 3.07 ± 0.59 preoperatively to 1.20 ± 0.86, and mean HFC grade improved from 1.07 ± 0.26 to 2.73 ± 0.88. Wilcoxon signed-rank tests confirmed significant improvements (MAS: *Z* = –3.42, *p* < 0.001, r = 0.88; HFC: *Z* =  + 3.35, *p* < 0.001, r = 0.87). Two patients with preoperative MAS grade 4 rebounded to grade 3, while the remaining 13 maintained a 2-grade reduction. The mean QuickDASH score improved from 52.47 ± 6.01 to 34.00 ± 7.97 (*p* < 0.001, d = 2.56), and the mean Barthel Index increased from 66.73 ± 4.30 to 83.00 ± 5.26 (*p* < 0.001, d = 3.42). All improvements were statistically significant and showed large effect sizes.

**Conclusions:**

T1 rhizotomy appears safe and effective for post-stroke hand flexion spasticity, significantly reducing spasticity and improving hand function. Further studies with extended follow-up are warranted.

## 1 Introduction

Central upper limb spasticity is a common sequela of brain or spinal cord injury, characterized by increased muscle tone and hyperactive tendon reflexes. Its typical presentation includes shoulder adduction and internal rotation, elbow flexion, forearm pronation, wrist flexion, finger flexion, and thumb-in-palm deformity (1). This spastic state severely impairs patients’ quality of life and, if progressive, can lead to joint stiffness and tendon contractures, resulting in permanent functional impairment (2, 3). Since upper limb function is ultimately expressed through hand use, flexion spasticity of the hand often causes significant loss of manual dexterity. Therefore, effective management of central upper limb spastic paralysis relies heavily on alleviating hand flexion spasticity.

Management options for central hand flexion spasticity to reduce muscle tone include rehabilitation therapy, pharmacological treatment, and surgical intervention (1, 4, 5). In severe cases, various surgical procedures have been explored, such as brachial plexus dorsal rhizotomy (6), selective posterior rhizotomy (7), C7 or C8 rhizotomy (8, 9), and contralateral C7 nerve transfer (10, 11). However, some of these techniques are associated with unsatisfactory recurrence rates over time (12).

Stroke is a leading cause of central upper limb spastic paralysis, accounting for a substantial proportion of clinical cases. Among post-stroke survivors, hand flexion spasticity is particularly common and disabling, as it directly compromises fine motor control, hygiene, and the ability to perform daily activities such as grasping and releasing objects. Despite the availability of various rehabilitation and pharmacological interventions, many patients with moderate-to-severe spasticity experience inadequate relief or intolerable side effects, and surgical options currently employed—such as selective posterior rhizotomy or contralateral C7 nerve transfer—are either technically demanding, associated with significant morbidity, or prone to recurrence of spasticity over time. Notably, T1 rhizotomy has been proposed as a potentially simpler and more targeted approach to reduce hand flexor tone, yet its safety and efficacy remain insufficiently validated in well-defined patient populations. To address this gap, the present study innovatively focuses on post-stroke patients with hand flexion spasticity—the most prevalent clinical subgroup—and systematically investigates the safety and efficacy of T1 rhizotomy, thereby providing evidence to support its clinical application in this challenging condition.

## 2 Methods

### 2.1 Study population

Between November 2023 and December 2024, 15 consecutive patients with post-stroke hand flexion spasticity underwent T1 rhizotomy at Jinan Third People's Hospital (China) in this uncontrolled, single-arm, single-center case series (Level IV evidence). The cohort included 12 males and 3 females, aged 34 to 72 years (mean 53.2 years). Ten cases were due to cerebral hemorrhage and five to cerebral infarction. The interval from spasticity onset to surgery ranged from 1 to 6 years (mean 3.01 ± 1.63). Surgical indications were as follows: (i) Adults with unilateral central hand flexion spasticity following a stroke, with a disease duration of ≥12 months and a functional plateau despite systematic medical and rehabilitation therapy (excluding botulinum toxin injections, neurolysis, or other surgical procedures). (ii) hand function assessment meeting the following criteria: finger flexor muscle tone graded ≥ 2 on the Modified Ashworth Scale (MAS) (13), with finger flexor muscle strength ≥ grade 2, and absence of fixed joint deformities secondary to contractures of the finger flexors, wrist flexors, or thumb adductors. The study protocol was approved by Ethics Committee of Jinan Third People's Hospital [approval No. (2023-KF-056)]. Informed consent was obtained from all participants.

To ensure homogeneity of the study population and to facilitate intraoperative localization of the target nerve root, all enrolled patients underwent standardized preoperative evaluations. Brachial plexus magnetic resonance neurography and ultrasonography were performed to assess the course and length of the supraclavicular brachial plexus nerve roots and trunks, with particular focus on the C8 and T1 nerve roots. These imaging data served as an anatomical reference for accurate and rapid identification of the T1 nerve root during surgery. Additionally, to minimize confounding effects on muscle tone assessment throughout the study period, all antispastic medications were maintained at constant dosages. Oral medications (baclofen and/or tizanidine) and botulinum toxin type A injections (if any) were continued at the same doses as before study enrollment, with no dose adjustments permitted during the observation period.

### 2.2 Assessment protocol and timeline

Follow-up was conducted for at least one year, during which spasticity was assessed using the MAS, and hand function was evaluated using the modified House Functional Classification (HFC) (14). To evaluate patient-reported functional recovery and activities of daily living, the QuickDASH (Disabilities of the Arm, Shoulder and Hand) questionnaire (15) was administered as a patient-reported outcome measure (PROM) preoperatively and at the final follow-up. In addition, the Barthel Index (16) was used to assess basic activities of daily living (ADL). All assessments were performed by an independent occupational therapist blinded to the preoperative imaging findings. The intraclass correlation coefficient (ICC) for MAS scoring was 0.93 (95% CI: 0.88–0.96). Assessments were conducted at the following time points: preoperative (within 1 week before surgery), postoperative day 1, day 3, week 2, month 1, month 3, month 6, and final follow-up (≥12 months).

### 2.3 Surgical procedure

**Figure 1:**
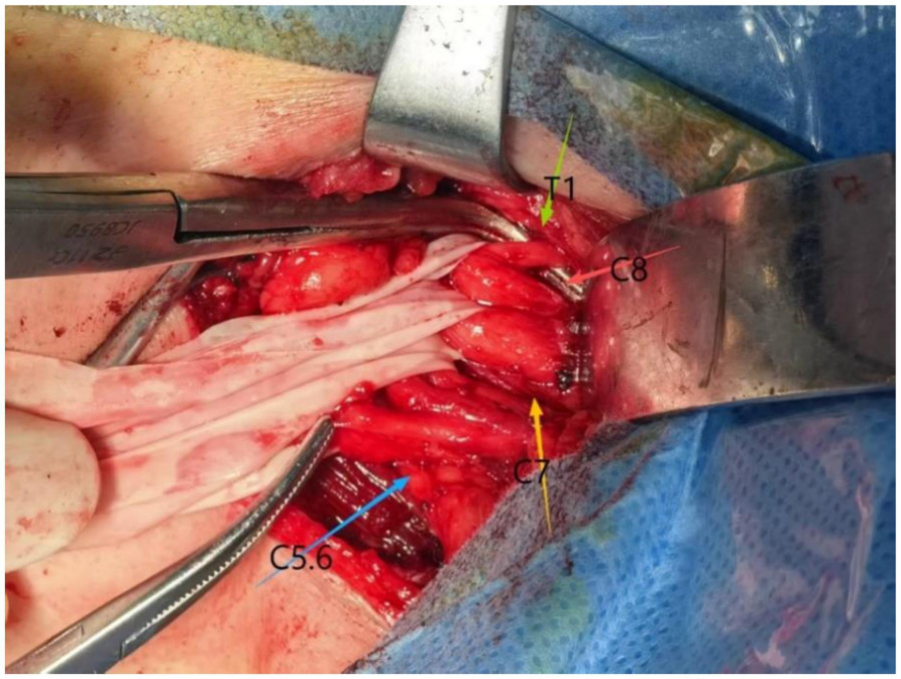
The T1 nerve root was exposed and identified.

**Figure 2:**
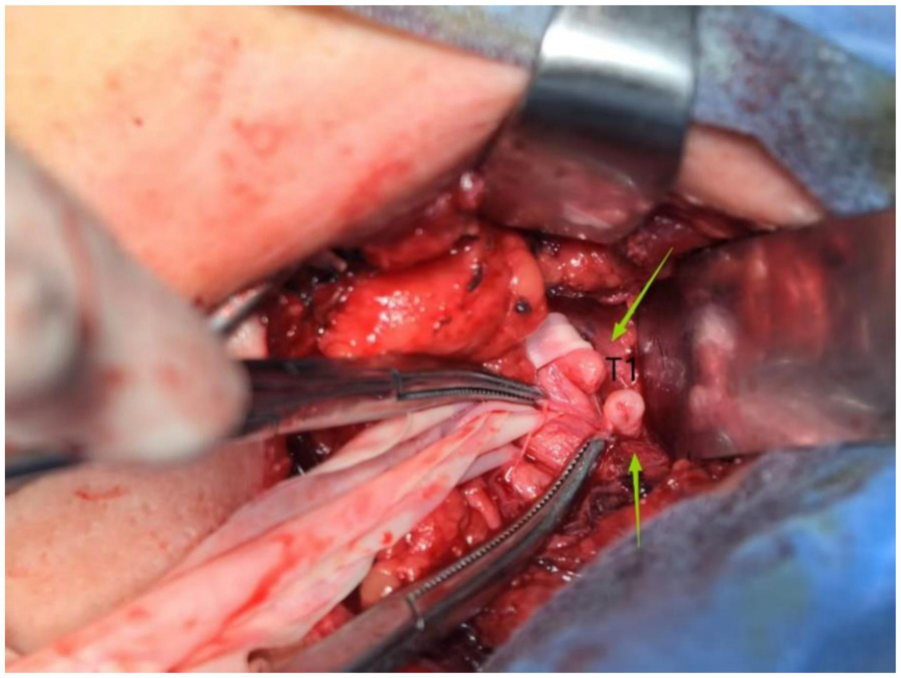
The T1 nerve root was transected.

General anesthesia with endotracheal intubation was administered. The patient was placed in a supine position. A transverse supraclavicular incision was made. Following incision of the skin and platysma muscle, the cutaneous nerves of the cervical plexus were preserved. The omohyoid muscle was retracted to expose the brachial plexus. Intraoperatively, the suprascapular nerve was first identified. Tracing it proximally led to the identification of the superior trunk, followed by the middle and inferior trunks. After locating the inferior trunk, the T1 nerve root was exposed by tracing the trunk further proximally (shown in Figure 1). Once the T1 root was identified, mechanical stimulation was applied to observe whether it elicited finger flexion. After performing a perineural lidocaine block and waiting for 2 min, the T1 nerve root was transected as distally as possible (shown in Figure 2). A segment approximately 1 cm in length was then excised from its proximal stump. The incision was closed in layers with interrupted sutures, and a drain or drainage strip was placed.

### 2.4 Statistical analysis

Continuous variables are expressed as mean ± SD. Normality was checked using the Shapiro–Wilk test. Wilcoxon signed-rank tests for ordinal variables (MAS, HFC), and Paired t-tests were used for normally distributed continuous variables (QuickDASH, Barthel Index). Effect sizes are reported as Cohen's d (for t-tests) or rank-biserial correlation r (for Wilcoxon tests).

## 3 Results

### 3.1 Surgical outcomes

The T1 rhizotomy was successfully completed by the same surgical team in all cases. The mean operative time was 50.33 min (range: 35–72 min), and the mean intraoperative blood loss was 12.4 mL (range: 5–35 mL). No iatrogenic vascular, neural, or visceral injury occurred.

### 3.2 Complications and adverse events

Postoperative complications included: delayed wound healing in one diabetic patient, resolved with dressing changes; a subcutaneous hematoma in one patient, improved after suture removal and drainage; and mild lymphatic leakage in one patient, resolved by postoperative day 7 with continuous negative pressure drainage.

On postoperative day 1, 3 patients experienced numbness in the little finger and medial forearm, which markedly reduced by one month and resolved within 3 months. Thirteen patients developed mild, tolerable forearm pain. Postoperative pain was further quantified using a 0–10 Visual Analog Scale (VAS); the mean VAS score on postoperative day 1 was 2.4 ± 1.1 (range 0–4). Pain was significantly relieved with oral gabapentin, diminishing by 6 weeks and resolving within 3 months.

All patients exhibited a postoperative decrease in finger flexor strength, which recovered to preoperative levels in most cases within 1–3 months. At final follow-up, no significant difference in muscle strength was observed compared to preoperative status.

### 3.3 Spasticity and hand function

On postoperative day 1, the MAS grades decreased by 1–3 grades in all patients. Improvement became more evident by day 3 and was maintained for 1.5–2 months. A rebound in tone was noted at 3 months in some cases with preoperative MAS grades 3–4. At final follow-up (≥12 months), two patients (both preoperative MAS grade 4) rebounded to grade 3, while the remaining patients maintained an average 2-grade reduction.

The mean MAS grade decreased from 3.07 ± 0.59 preoperatively to 1.20 ± 0.86 at final follow-up. A Wilcoxon signed-rank test confirmed that this reduction was statistically significant (*Z* = −3.42, *p* < 0.001, rank-biserial correlation *r* = 0.88), indicating a large effect size.

The modified HFC score improved by at least one grade at final follow-up in all patients. One patient improved from HFC grade 2 to 5, regaining independent grasp and partial functional hand use for daily life. The mean HFC grade improved from 1.07 ± 0.26 preoperatively to 2.73 ± 0.88 at final follow-up. A Wilcoxon signed-rank test showed a significant improvement (*Z* =  + 3.35, *p* < 0.001, *r* = 0.87), representing a large and clinically meaningful improvement in voluntary hand function.

### 3.4 Functional and ADL outcomes

**Figure 3:**
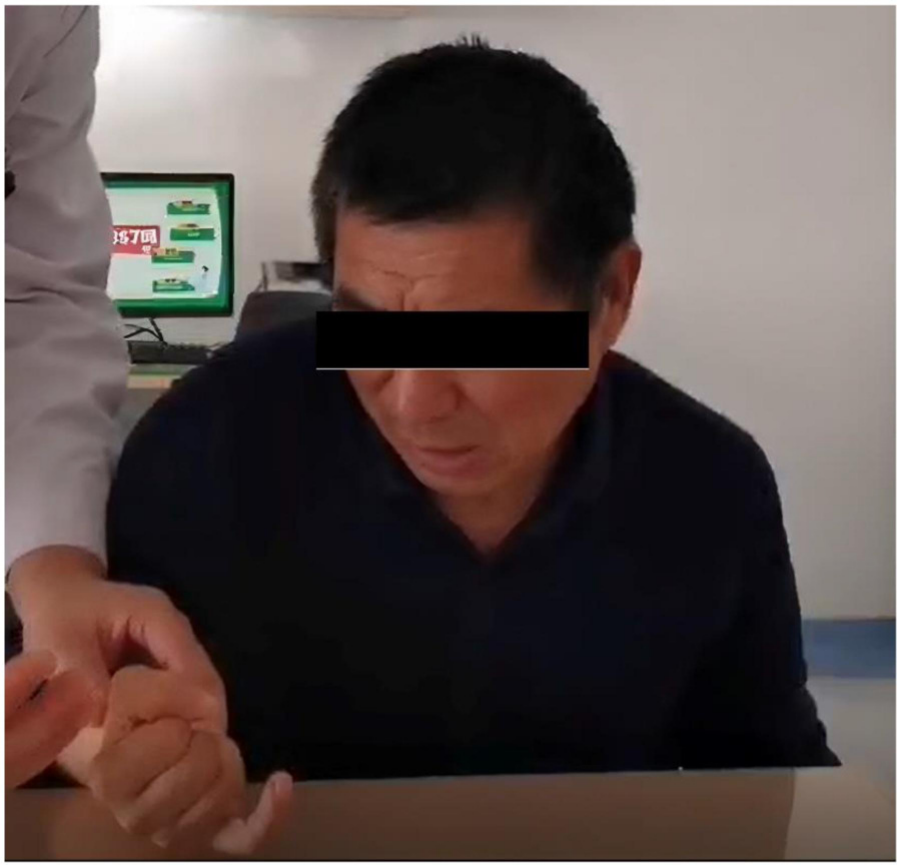
Preoperative hand performance of a 54-year-old male patient.

**Figure 4:**
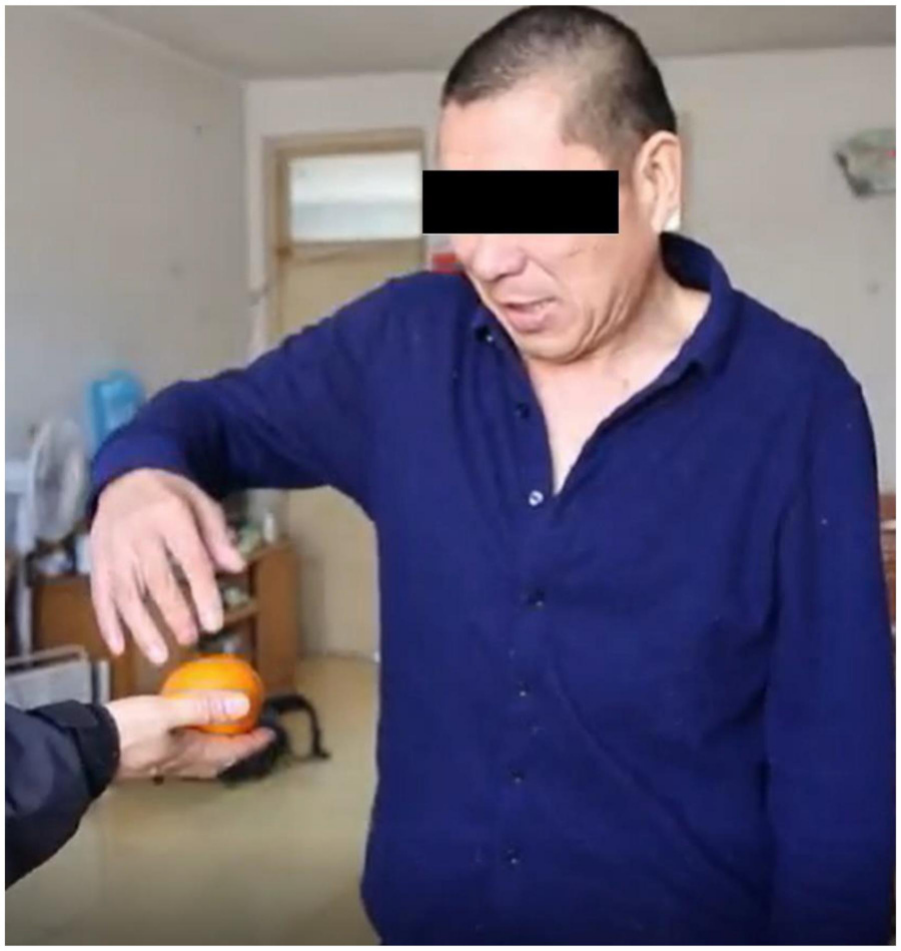
Postoperative hand performance at 12-month follow-up.

Functional outcomes assessed by the QuickDASH questionnaire improved significantly from a preoperative mean of 52.47 ± 6.01 to 34.00 ± 7.97 at final follow-up (paired t-test: mean difference = −18.47, 95% CI: −21.25 to −15.69, t = 21.87, *p* < 0.001, Cohen's d = 2.56). Activities of daily living measured by the Barthel Index increased from 66.73 ± 4.30 preoperatively to 83.00 ± 5.26 at final follow-up (mean difference = + 16.27, 95% CI: 13.13 to 19.41, t = 30.21, *p* < 0.001, Cohen's d = 3.42). These improvements indicate that T1 rhizotomy not only reduces spasticity and enhances hand function but also translates into better upper limb performance and greater independence in daily living. All effect sizes were large (Cohen's d > 0.8), confirming clinically meaningful improvements beyond statistical significance. Detailed clinical data are presented in Table 1. A representative case is shown in Figures 3, 4.

**Table 1:** Characteristics of the patients who underwent T1 rhizotomy.

Patient	Age	Gender	Cause	Duration（years）	Operative time (minutes)	Complications	MAS grade		HFC grade		QuickDASH		Barthel Index	
							Pre-op	Last FU	Pre-op	Last FU	Pre-op	Last FU	Pre-op	Last FU
1	60	M	CI	3	65	slight pain; numbness	4	3	1	2	56	42	65	78
2	51	M	CI	2	72	slight pain	3	1	1	2	48	30	70	85
3	46	M	CH	1	54	slight pain	3	1	2	5	52	28	68	88
4	48	M	CH	4	36	slight pain; numbness	3	1	1	3	58	35	62	80
5	34	M	CH	1.5	47	delayed wound healing	2	0	1	3	40	18	75	92
6	44	M	CH	6	58	slight pain	3	1	1	2	54	32	66	84
7	50	M	CH	3	50	slight pain; numbness	3	1	1	3	55	38	64	82
8	56	F	CH	4	36	slight pain; subcutaneous hematoma	4	3	1	2	62	48	60	72
9	68	M	CI	4	54	slight pain	4	2	1	2	60	44	61	76
10	63	M	CH	6	45	numbness; lymphatic leakage	3	1	1	3	57	40	63	80
11	48	M	CI	2.2	60	slight pain	3	1	1	3	50	34	68	84
12	72	F	CI	2	52	slight pain	2	0	1	4	44	22	72	90
13	56	F	CH	1	43	slight pain; numbness	3	1	1	3	53	36	67	83
14	54	M	CH	1.5	35	slight pain; numbness	3	1	1	2	47	30	71	86
15	51	M	CH	4	48	slight pain	3	1	1	2	51	33	69	85
Mean **±** SD	53.2 ± 9.78	-	-	3.01 ± 1.63	50.33 ± 10.73	-	3.07 ± 0.59	1.2 ± 0.86	1.07 ± 0.26	2.73 ± 0.88	52.47 ± 6.01	34.00 ± 7.97	66.73 ± 4.30	83.00 ± 5.26

M, male; F, female; CI, cerebral infarction; CH, cerebral hemorrhage; MAS, Modified Ashworth Scale; HFC, House Functional Classification; Pre-op, preoperative; Last FU, last follow-up.

## 4 Discussion

### 4.1 Pathophysiology of spasticity

Spasticity is a velocity-dependent increase in tonic stretch reflexes, characteristic of upper motor neuron syndrome (17). Following an upper motor neuron lesion, loss of supraspinal inhibitory inputs—primarily from the corticospinal, reticulospinal, and dorsal reticulospinal tracts—results in persistent hyperexcitability of spinal *α*- and *γ*-motor neurons (18).

At the neurotransmitter level, an imbalance between inhibitory and excitatory transmission underlies spasticity. Spinal GABA (gamma-aminobutyric acid) is relatively deficient after cortical or spinal injury, leading to reflex disinhibition (17). Glutamate excitotoxicity during the post-injury period further leads to overactivation of glutamate receptors and downregulation of GABAergic inhibitory tone, as documented in both spinal cord injury and stroke models (19, 20). Glycine, another spinal inhibitory transmitter, together with GABA mediates reciprocal inhibition between agonist and antagonist muscles (21). Reduced glycinergic inhibition may impair reciprocal inhibition essential for coordinated movement, potentially contributing to co-contraction in spastic patients.

At the muscle level, chronic spasticity induces secondary structural adaptations. Increased collagen deposition in the extracellular matrix of spastic muscles correlates positively with stiffness (22). Intramuscular connective tissue remodeling leads to fibrosis and reduced elasticity (23). Additionally, sarcomeres in spastic muscle are reportedly excessively long, contributing to poor force generation and weakness (5). These neural and non-neural changes imply that spasticity involves not only hyperreflexia but also irreversible muscle structural alterations (23).

The tendon also adapts in chronic spasticity. Tendon mechanical properties depend largely on collagen cross-linking, which is sensitive to mechanical loading (24). Prolonged unloading and abnormal loading in spastic limbs may alter cross-linking profiles, reducing collagen fiber alignment and elasticity, thus limiting joint range of motion. Although direct studies on tendon adaptation in spastic patients are limited, experimental evidence suggests that altered cross-linking directly affects tendon mechanics (24). These pathophysiological insights provide the rationale for nerve-targeted surgical interventions, such as T1 rhizotomy, which aim to interrupt hyperexcitable spinal circuits at the most affected segment (25).

### 4.2 Current surgical landscape

Stroke is a common cause of central upper limb spasticity, affecting 17%–43% of patients (26). Reducing the muscle tone of the finger flexor muscles in the affected hand is a prerequisite for restoring and reconstructing hand function. While various non-surgical options exist, surgery remains indicated for refractory cases. As emphasized by Gart et al. (27), surgical interventions must be highly individualized. Prior approaches—such as brachial plexus dorsal rhizotomy (effective in pediatric cerebral palsy) (6), C7 or C8 rhizotomy (short-term relief with frequent recurrence) (9), and contralateral C7 transfer (10, 11) (suboptimal long-term outcomes)—have limitations.

### 4.3 Clinical experience with T1 rhizotomy

The team led by Professor Shufeng Wang at Beijing Jishuitan Hospital first proposed T1 rhizotomy for central hand flexion spasticity (28). As a participating center, our hospital has performed >50 procedures since November 2023, with the longest follow-up reaching two years. In our cohort, short-term efficacy was notable, especially within the first two months. At final follow-up, 13 patients maintained a 2-grade MAS reduction, indicating sustained tone reduction. Consequent improvement in hand flexion-extension and modified HFC grade was observed.

### 4.4 Rationale for T1 rhizotomy

The precise mechanism by which T1 rhizotomy reduces spasticity and improves hand function remains incompletely understood. Severity of muscle hypertonia correlates with the extent of upper motor neuron injury: greater damage to specific motor cortical areas results in more pronounced disinhibition of spinal *γ*-motor neurons. An understanding of the anatomical and neurophysiological basis for targeting T1 rather than C8 is essential. Anatomically, intrinsic hand muscle innervation originates predominantly from the C8 and T1 spinal nerve roots, with T1 playing a dominant role (29, 30). The T1 root contributes a larger proportion of fibers to the median and ulnar nerves, which supply the finger flexors and most intrinsic hand muscles, compared to the C8 root (31, 32). Following an upper motor neuron lesion, *γ*-motor neuron hyperexcitability is not uniform across spinal segments; it is more pronounced in segments with stronger cortical representation and greater reliance on descending modulation. Although direct comparative imaging evidence for differential cortical representation of T1 versus C8 is lacking, functional imaging studies have established that hand muscles have a well-defined representation in the primary motor cortex (33, 34). Given that the T1 root provides dominant innervation to the median-nerve-supplied finger flexors and most intrinsic hand muscles, it is plausible that after a widespread cortical or subcortical stroke, the disinhibition of *γ*-motor neurons in the T1 spinal segment may exceed that in C8, making T1 a critical contributor to finger flexion spasticity. Selective transection of the T1 root interrupts the afferent limb of the *γ*-loop at the most hyperexcitable segment, thereby reducing muscle spindle sensitivity and dampening *α*-motor neuron output to the finger flexors (25, 35). In contrast, C8 rhizotomy alone may leave a substantial portion of the hyperexcitable T1-driven input intact, likely resulting in less complete or less durable spasticity relief. This neuroanatomical rationale supports T1 as the primary surgical target for post-stroke hand flexion spasticity.

### 4.5 Rebound spasticity: observations and hypotheses

Two patients with preoperative MAS grade 4 experienced rebound: their scores dropped from 4 to 3 postoperatively but remained at 3 at final follow-up (net reduction of only one grade). Higher preoperative tone may predispose to recurrence. Possible mechanisms include: (1) incomplete denervation—C8 root remains intact and may compensate; (2) central neuroplasticity—motor cortex reorganizes to amplify descending inputs to residual spinal circuits; (3) peripheral reinnervation from adjacent nerve sprouts (less likely with proximal root transection). Clinically, T1 rhizotomy alone may be insufficient for the most severe (MAS 4) spasticity. A multimodal approach combining rhizotomy with orthopedic procedures (e.g., flexor tendon release or transfer) may be necessary for sustained improvement.

### 4.6 Limitations and future directions

This study has several limitations. First, MAS is a subjective ordinal scale with inherent interobserver variability; although assessments were performed by the same two trained physicians, subjectivity persists. Future studies should include objective modalities (electrophysiological or biomechanical). Second, this single-center study had a small sample size (*n* = 15), limiting generalizability. The lack of a control group or comparison with other techniques (e.g., C8 rhizotomy, contralateral C7 transfer) constrains conclusions on relative efficacy. Third, follow-up (≥12 months) may be insufficient to capture late recurrences; longer follow-up is needed. Fourth, no objective electrophysiological (e.g., EMG, H-reflex) or biomechanical (torque-controlled) quantification of spasticity reduction was performed. Additionally, we did not evaluate psychological outcomes, caregiver burden, patient satisfaction, or cost-effectiveness. Finally, selection bias cannot be excluded, as all patients were recruited from a single tertiary center with uniform rehabilitation.

Current surgical approaches for upper limb spasticity target peripheral muscle, tendon, and joint manifestations without addressing underlying neuropathology, leading to unsatisfactory recurrence over time (12). Thus, no single optimal technique exists. Our initial experience is encouraging, but limitations (single-center, small sample, no comparator, limited long-term follow-up) remain. Future multicenter randomized controlled trials are warranted. Looking forward, novel surgical strategies combined with brain–computer interfaces and robotics may transform treatment of central spastic paralysis.

## 5 Conclusion

T1 rhizotomy is a safe and effective surgical option for post-stroke hand flexion spasticity, providing significant and sustained reductions in muscle tone along with improvements in hand function and activities of daily living. The procedure is associated with minimal, transient complications. However, patients with preoperative MAS grade 4 may experience spasticity rebound, suggesting that T1 rhizotomy alone might be insufficient for the most severe cases; a combination with orthopedic procedures should be considered. Given the study's limitations, larger multicenter randomized controlled trials with longer follow-up and objective outcome measures are warranted to validate these findings.

## Data Availability

The original contributions presented in the study are included in the article/Supplementary Material, further inquiries can be directed to the corresponding author.
